# Measuring private equity penetration and consolidation in emergency medicine and anesthesiology

**DOI:** 10.1093/haschl/qxad008

**Published:** 2023-06-20

**Authors:** Loren Adler, Conrad Milhaupt, Samuel Valdez

**Affiliations:** Brookings Institution, 1775 Massachusetts Avenue NW, Washington, DC 20036, United States; Brookings Institution, 1775 Massachusetts Avenue NW, Washington, DC 20036, United States; University of Southern California, 2250 Alcazar St, Los Angeles, CA 90007

**Keywords:** surprise billing, emergency medicine, anesthesiology, private equity, balance billing, consolidation, antitrust

## Abstract

Private equity–backed staffing companies in anesthesia and emergency medicine, as well as those owned by publicly traded companies, gained notoriety for driving surprise billing—a practice where patients unexpectedly treated by an out-of-network provider can be billed for the difference between the provider's charge and what their insurer pays. Yet, little is known about the evolution of private equity and publicly traded company investment in these specialties. In this study, we construct a novel dataset identifying the ownership structure of anesthesia and emergency medicine physician groups to document trends in consolidation and the growing role of private equity and publicly traded companies. From 2009 to 2019, we found substantial increases in local market concentration in each specialty and that physician groups owned by private equity or publicly traded companies grew from 3.2% and 8.6% of the national anesthesia and emergency medicine markets, respectively, to 18.8% and 22.0%.

## Introduction

Private equity (PE) investment in physician practices continues to grow rapidly and capture the attention of policymakers. The Federal Trade Commission (FTC) has indicated an interest in tracking and studying the PE model of acquiring dozens of small medical practices that each individually evade antitrust scrutiny but, taken together, generate high levels of local market concentration. And the FTC recently opened an investigation into the market power of US Anesthesia Partners, one of the largest PE-owned anesthesiology practices in the country.^1^

Large anesthesia and emergency medicine staffing companies owned by PE or publicly traded companies also gained notoriety for leveraging surprise out-of-network billing, a practice whereby consumers can be directly billed for the difference between their provider's charge and what their insurer pays. Empirical evidence suggests that these staffing companies increased the prices paid by commercial insurers and patients for anesthesia and emergency physician services.^2,3^ However, relatively little is known about the extent of investment by PE and publicly traded companies in these specialties, how fast it grew as surprise billing started to generate more media attention in the 2010s, or how much a decade of dealmaking increased consolidation in anesthesia and emergency medicine markets.

Hospitals and other medical facilities can elect to either staff their own emergency rooms and anesthesia departments or contract with an outside company or medical group for staffing. Public reporting suggests that about one-third of hospitals employ their own emergency medicine and anesthesia professionals, but less is known about the ownership breakdown of contracted groups.^4^ Many facilities contract with large staffing companies owned by PE firms (some of which have cycled back and forth between PE ownership and being publicly traded over time) for these specialty services, while others contract with independently owned medical groups or staffing companies.

In this study, we create a novel dataset identifying the parent company and ownership structure of physician groups from 2009 to 2019 and combine it with Medicare data to characterize the penetration (ie, market shares) of PE and publicly traded companies in anesthesia and emergency medicine, as well as local market concentration levels in these specialties.

This research can help inform regulatory agencies seeking to better understand market concentration in these specialties and lay the groundwork for future research into the effects of PE or publicly traded company acquisitions and broader physician consolidation.

## Study data and methods

We constructed a longitudinal dataset from 2009 to 2019 that identified the parent company and ownership structure of medical groups—as defined by their taxpayer identification number—that billed Medicare for emergency medicine or anesthesia services at the end of each calendar year. This dataset was then combined with Medicare claims data to calculate the market shares of different medical groups and the combined market shares of groups owned by PE or a publicly traded company. We generally considered PE and publicly traded companies together in this analysis because some of the largest staffing companies alternated between these ownership structures during our study period, and because some of the PE firms themselves are publicly traded.

Specifically, we began with the Medicare Fee-For-Service claims data published by the Centers for Medicare and Medicaid Services. These data comprised all Medicare Part B claims for our study period of 2009 through 2019. We restricted our analytic sample to claims for anesthesia (current procedural terminology [CPT] codes 00100–01999) and emergency medicine (CPT codes 99281–99285, 99291–99292) services. We excluded any claims where (1) Medicare was not the primary payer, (2) the claim was denied, (3) the claim had nonpositive allowed amounts, or (4) the service for the claim was not rendered in the fifty states or District of Columbia.

Next, we constructed an extensive crosswalk that mapped medical groups in the Medicare claims data to their parent companies and identified the ownership structure of the parent company. The crosswalk combined information from the Medicare Data on Provider Practice and Specialty dataset, Medicare Care Compare: Doctors and Clinicians national files, National Plan and Provider Enumeration System data, Pitchbook deals data, articles of incorporation documents from state-level Secretary of State websites, company websites, news articles, and Google searches.^5–8^ For a detailed description of our crosswalk methodology, see supplementary appendix A.

In order to measure market concentration, we incorporated data from the Dartmouth Atlas of Health Care to identify the hospital referral regions (HRRs) where each claim was delivered.^9^ Market shares for each parent company were calculated as the sum of their Medicare allowed amounts for all emergency medicine or anesthesia services in a given year divided by the total Medicare allowed amounts for those services delivered in the HRR. We then calculated the Herfindahl-Hirschman index (HHI) for each HRR in each year. We adopted the Department of Justice definitions of concentration based on each market's HHI and used data from the Integrated Public Use Microdata Series (IPUMS) National Historical Geographic Information System to weight HRRs by population.^10,11^

## Limitations

The main limitation is that our data come only from the Medicare market. If significant differences in market composition exist between the Medicare and commercial markets, our estimates of market shares and concentration levels may not be generalizable to the commercial market. However, emergency medicine and anesthesia groups typically contract with a hospital to treat patients from all payers, so we expect smaller differences between Medicare and commercial market concentration measures than might exist for office-based medical specialties.

While the process we undertook to identify the ownership of medical groups was extensive and included substantial manual checks, it is possible that we missed some acquisitions by PE or publicly traded companies. However, our estimates are similar to publicly available data on the market shares of specific companies in a given year or in a specific location (see supplementary appendix A).

## Study results

**Figure 1.**
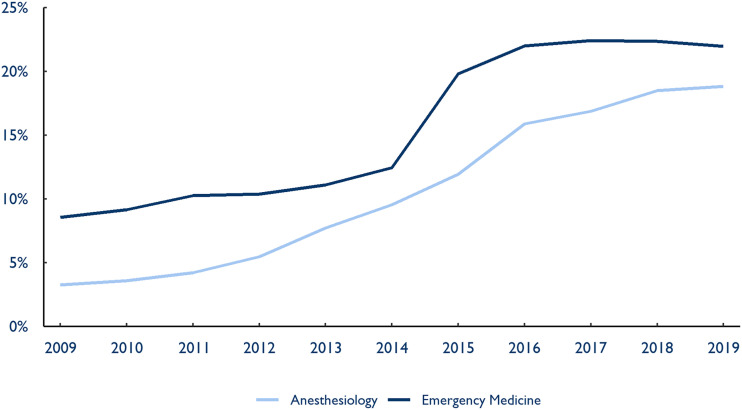
National market share of private equity and publicly traded companies, anesthesiology and emergency medicine, 2009–2019. Source: Authors’ analysis of novel dataset identifying the parent company and ownership type of medical groups in the 2009–2019 Medicare Fee-For-Service claims data.

In 2009, PE and publicly traded companies controlled 3.2% and 8.6% of the national anesthesia and emergency medicine markets, respectively. By 2019, however, their presence had grown to 18.8% of the anesthesia market and 22.0% of the emergency medicine market nationally, with a striking acceleration between 2014 and 2016 (Figure 1).

**Figure 2.**
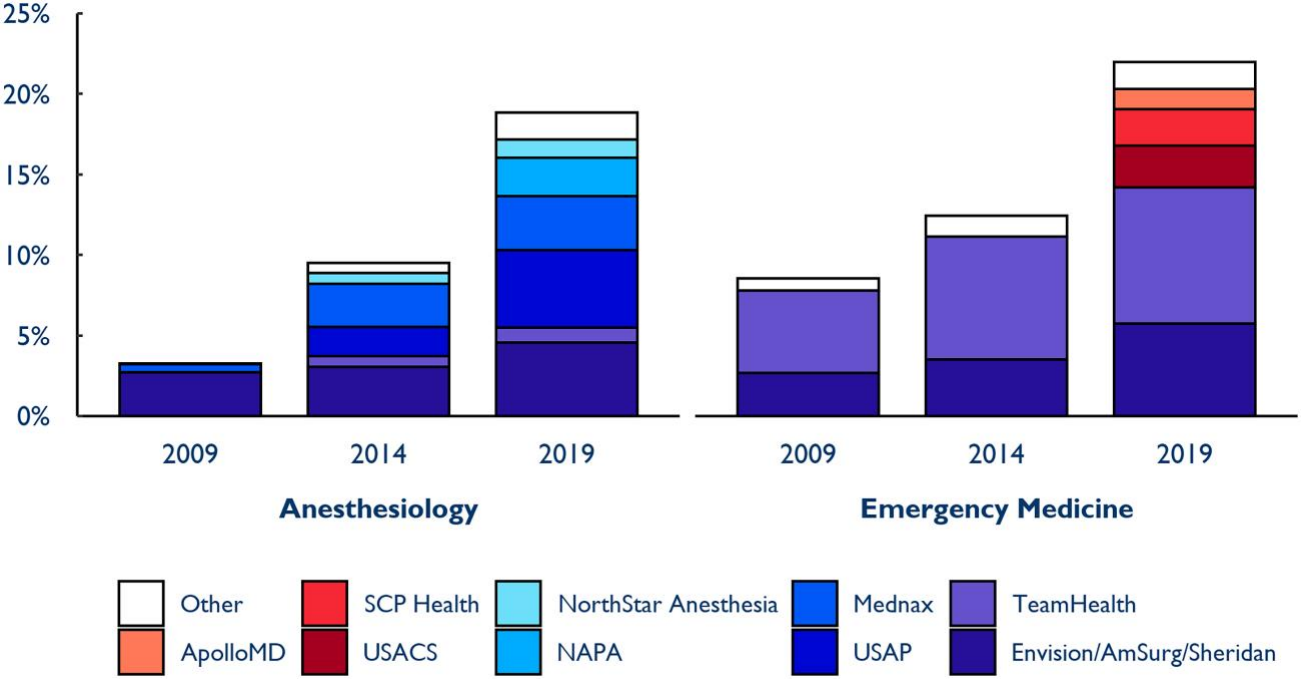
Largest staffing companies owned by private equity or publicly traded parent companies by national market share, 2009–2019. The figure reports the names of the underlying staffing companies. USAP = U.S. Anesthesia Partners; NAPA = North American Partners in Anesthesia. For more detailed information on the ultimate controlling parent companies, see supplementary appendix B. Source: Authors’ analysis of novel dataset identifying the parent company and ownership type of medical groups in the 2009–2019 Medicare Fee-For-Service claims data.

Each of the five largest independent anesthesia practices in 2009 and five of the six largest independent emergency medicine practices were acquired by PE or publicly traded companies over our study period. Existing companies such as TeamHealth, Mednax, and Envision grew larger in the decade that we studied, and many new entrants gained significant market share by 2019 (Figure 2).

For example, US Anesthesia Partners (USAP) started out as the platform company established by the PE firm Welsh, Carson, Anderson, & Stowe (WCAS) when they acquired Greater Houston Anesthesiology, which at the time was the second largest independent company (0.6% of the national market in 2012, the year prior to acquisition). After acquiring other medical groups, USAP became the single largest anesthesia parent company by 2019, controlling 4.8% of the national market. A similar strategy was pursued in emergency medicine, where WCAS acquired the second largest independent company in 2015 to form US Acute Care Solutions. It became the fourth largest emergency medicine parent company by 2019 (2.6% of the national market), competing with TeamHealth and Envision, which controlled 8.5% and 5.8%, respectively, of the national emergency medicine market that year.

While large platform acquisitions represented the largest transfers of market share from independent practices to PE, they had no direct effect on local market concentration levels. However, platform acquisitions were typically followed by a series of add-on acquisitions, many in the same geographic markets, which increased concentration. Further consolidation was also spurred by a series of mergers and acquisitions over our study period among hospitals who employed their anesthesia and/or emergency medicine practitioners.

In 2009, we estimate that 59.8% of Americans lived in an HRR with an anesthesia market that was unconcentrated as defined by the Department of Justice (ie, an HHI below 1500). Similarly, 64.6% of the country lived in an HRR with an unconcentrated emergency medicine market. Only about one-sixth of the US population lived in HRRs that were highly concentrated (defined as an HHI greater than 2500) in either specialty, where competition is greatly diminished and regulators typically apply greater scrutiny to further consolidation.

**Figure 3.**
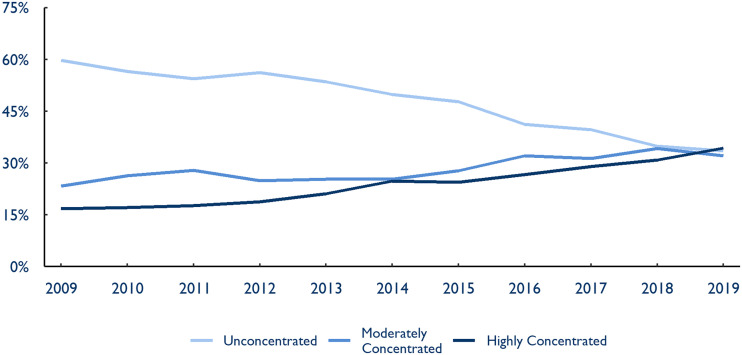
Share of US population by hospital referral region (HRR) concentration level, anesthesiology, 2009–2019. Concentration levels are reported using Department of Justice definitions of market concentration. An HRR with a Herfindahl-Hirschman index (HHI) below 1500 is considered unconcentrated, between 1500 and 2500 moderately concentrated, and above 2500 highly concentrated. Each HRR is then weighted by population using IPUMS National Historical Geographic Information System data to report metrics as a share of the US population. Source: Authors’ analysis of novel dataset identifying the parent company and ownership type of medical groups in the 2009–2019 Medicare Fee-For-Service claims data.

But, by 2019, the share of the population living in a highly concentrated anesthesia market more than doubled from 16.8% to 34.4% (Figure 3). The share of the population living in a moderately concentrated anesthesia market (an HHI between 1500 and 2500) also increased to 32.1% from 23.4%. Only 33.5% of Americans resided in an unconcentrated market by 2019—nearly cut in half since 2009.

**Figure 4.**
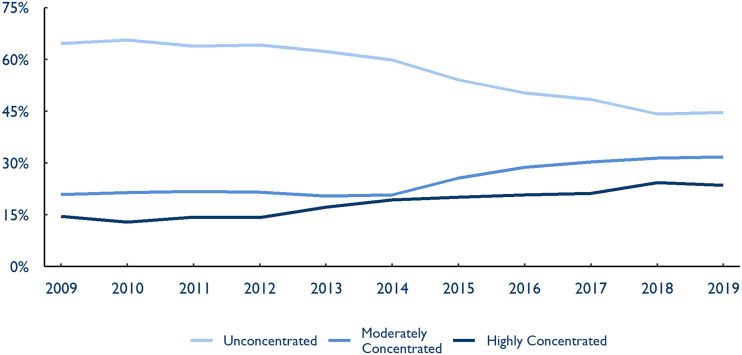
Share of US population by hospital referral region (HRR) concentration level, emergency medicine, 2009–2019. Concentration levels are reported using Department of Justice definitions of market concentration. An HRR with a Herfindahl-Hirschman index (HHI) below 1500 is considered unconcentrated, between 1500 and 2500 moderately concentrated, and above 2500 highly concentrated. Each HRR is then weighted by population using IPUMS National Historical Geographic Information System data to report metrics as a share of the US population. Source: Authors’ analysis of novel dataset identifying the parent company and ownership type of medical groups in the 2009–2019 Medicare Fee-For-Service claims data.

Similar, albeit less drastic, changes took place in the emergency medicine market (Figure 4). Following three large acquisitions by PE firms in 2015 and a series of follow-on deals, the share of Americans living in an unconcentrated emergency medicine market fell from 59.8% in 2014 to 44.7% in 2019. The share of Americans living in both moderately and highly concentrated markets increased by more than 50% between 2009 and 2019.

For context, a study of various office-based specialty markets in the United States found that 22% were highly concentrated in 2013 and 21% were moderately concentrated.^12^ As an unweighted share of HRRs (the closest comparable metric despite differences in market definition), we found that 71% of anesthesia markets and 68% of emergency medicine markets were highly or moderately concentrated in 2013. These numbers grew to 84% and 80% in anesthesia and emergency medicine, respectively, by 2019. This suggests that concentration levels in these two specialties already exceeded those in other specialties as of 2013, and that high or moderate market concentration became even more common with time.

**Figure 5.**
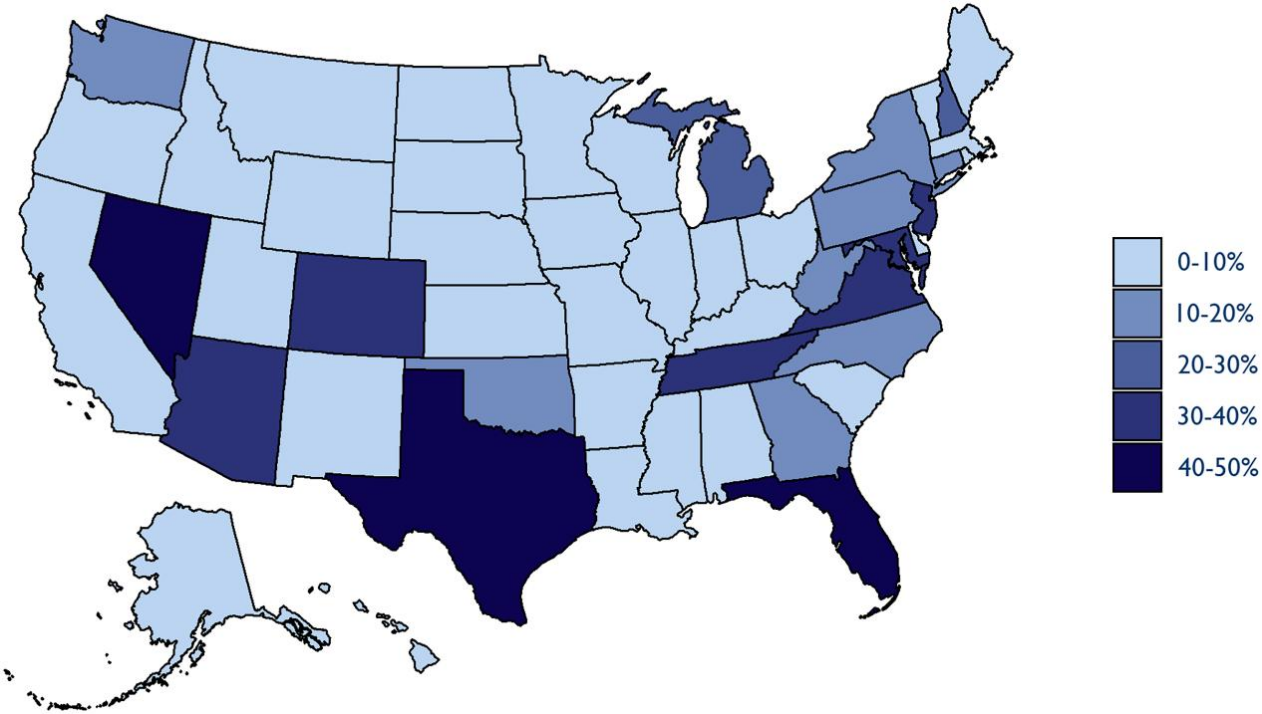
Private equity and publicly traded company market share, anesthesiology (2019). Source: Authors’ analysis of novel dataset identifying the parent company and ownership type of medical groups in the 2009–2019 Medicare Fee-For-Service claims data.

We also found that the market share accounted for by PE and publicly traded companies in anesthesia and emergency medicine varied substantially across the country. In the anesthesia market, these companies were especially active in Florida, Texas, Nevada, and Washington, DC—more than 40% of each state market was controlled by PE and publicly traded companies in 2019. Parts of the Mid-Atlantic and New England also saw significant investment from PE or publicly traded companies, as did Arizona, Colorado, and Tennessee (Figure 5).

In several states, a single PE-owned parent company controlled a quarter or more of the anesthesia market. In 2019, USAP controlled 30% of the Nevada and Colorado markets, and 36% of the Texas market. The large PE firm Kohlberg, Kravis, Roberts, which took Envision private in 2018, controlled 23% of the Arizona market, 27% of the Florida market, and 19% of the New Jersey market at the end of our study period.

**Figure 6.**
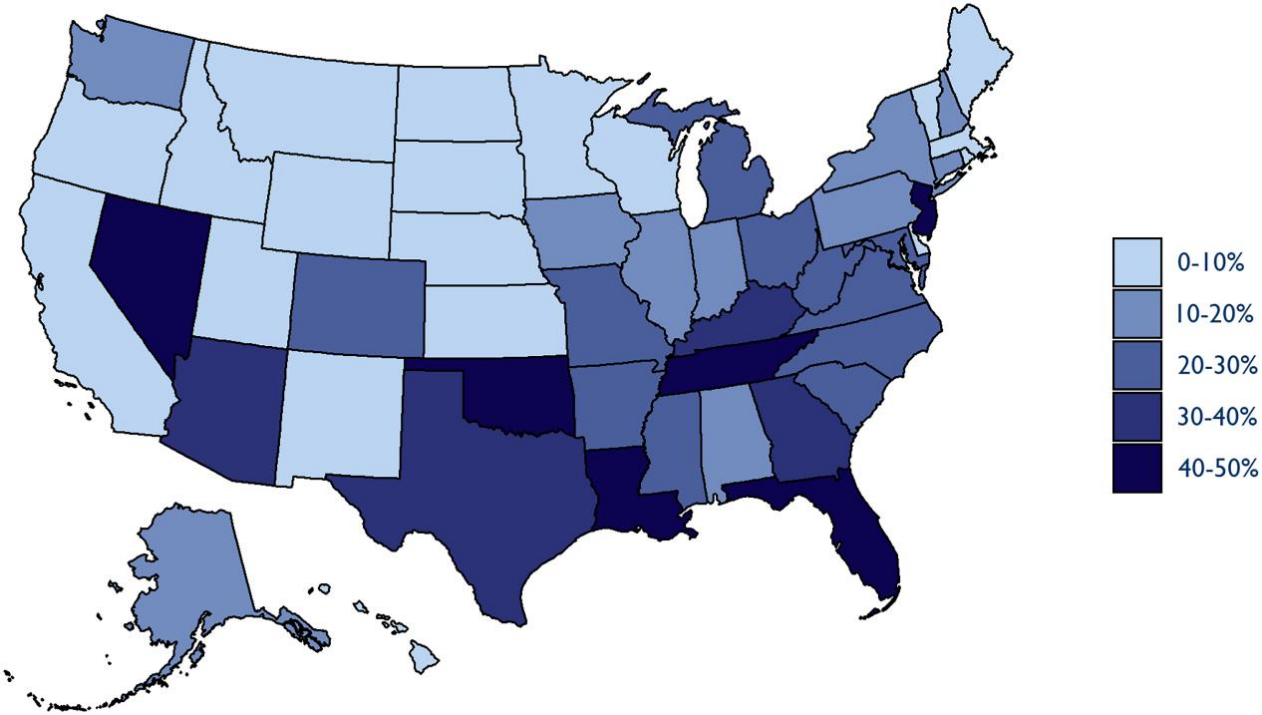
Private equity and publicly traded company market share, emergency medicine (2019). Source: Authors’ analysis of novel dataset identifying the parent company and ownership type of medical groups in the 2009–2019 Medicare Fee-For-Service claims data.

Investment from PE and publicly traded companies was even more widespread in the emergency medicine market (Figure 6). In 2019, there were twenty-one states with more than 20% of the emergency medicine market controlled by these companies compared with just twelve such states in the anesthesia market. Similarly, fewer states were untouched by PE or publicly traded company investment in emergency medicine compared with anesthesia. There were twenty states with less than 10% of the emergency medicine market controlled by PE and publicly traded companies compared with thirty-one such states in anesthesia.

State dominance by a single PE company was also more common in the emergency medicine market. In eight states, a single PE parent company controlled more than 20% of the market. And the PE firm Blackstone, which acquired TeamHealth in 2017, controlled 15% or more of the emergency medicine market in Florida, Kentucky, Nevada, New Jersey, Oklahoma, South Carolina, Tennessee, Washington, and West Virginia.

Map graphics depicting the geography of unconcentrated, moderately concentrated, and highly concentrated HRRs can be found in supplementary appendix C. Summary tables of the largest parent companies in the most highly concentrated HRRs can be found in supplementary appendix D.

## Discussion

Our findings demonstrate that PE and publicly traded companies have become a major force in both the anesthesia and emergency medicine markets over the last decade. We estimate that they controlled 18.8% and 22.0% of the national anesthesia and emergency medicine markets, respectively, in 2019—a sixfold increase in anesthesia and nearly a threefold increase in emergency medicine since 2009. This rapid expansion was driven both by large platform acquisitions and a series of smaller add-on deals and was especially pronounced in certain states such as Florida, Nevada, Tennessee, and Texas. PE and publicly traded company acquisitions, along with hospital mergers that increased consolidation, also substantially reduced competition in many local anesthesia and emergency medicine markets across the United States.

Since 2019, several large acquisitions have further reduced competition in these markets. For instance, North American Partners in Anesthesia (NAPA)—owned by the PE firm American Securities—was the fourth largest anesthesia company in 2019, controlling 2.4% of the national market. In May of 2020, NAPA acquired American Anesthesiology, a subsidiary of Mednax (the third largest anesthesia company in 2019, representing 3.3% of the national market). By our calculations, this deal made NAPA the largest anesthesia company in the country, increasing market concentration in some large HRRs. For example, the estimated combined market share of the two companies in 2019 was 71.9% in the Newark, New Jersey, HRR and 82.1% in the Arlington, Virginia, HRR.

It is notable that the rapid growth of PE and publicly traded company ownership in anesthesia and emergency medicine—the two specialties most linked to surprise out-of-network billing—occurred alongside growing interest from state and federal policymakers in protecting patients from surprise bills, eventually culminating in passage of the federal No Surprises Act at the end of 2020. While our study does not provide causal evidence, other research suggests that large staffing companies owned by PE or publicly traded companies increased commercial prices and the prevalence of out-of-network billing.^2,3^

The trends we document raise concerns given the strong evidence that both horizontal consolidation in physician markets generally—and PE acquisitions in particular—lead to higher commercial insurance prices.^2,3,13–15^ Little is known, though, about the effects of PE investment on the quality of care. More research is therefore needed to better understand the effects of physician practice acquisitions on both quality and costs.

## Supplementary Material

qxad008_Supplementary_Data
