# Supplementary material for: Measuring private equity penetration and consolidation in emergency medicine and anesthesiology
Source: Health Aff Sch. 2023 Jun 20;1(1):qxad008. doi: 10.1093/haschl/qxad008 (PMC10986250; doi:10.1093/haschl/qxad008)
Supplement: qxad008_Supplementary_Data [file qxad008_Supplementary_Data.zip › PE in EM Anesthesia Appendices (for R&R).docx]

**Appendix A. Mapping Medical Groups to Their Parent Companies and Ownership Structures**

At the core of our analysis is a newly constructed longitudinal dataset from 2009 to 2019 that identifies the parent company and ownership structure of medical groups, as defined by their taxpayer identification number (TIN), as of the end of each calendar year. A TIN is a billing entity that captures a group of health professionals who practice under common ownership, but in many cases, a single parent company can operate under numerous TINs.^[[1]](#footnote-2)^ Neither common ownership of TINs nor their ownership structure (e.g., private equity, publicly-traded company, health system, independent private company) is captured in existing data sources.

To build this dataset, we began with the Medicare Fee-For-Service claims data published by the Centers for Medicare and Medicaid Services (CMS). Each claim-level observation includes key information including the rendering provider’s national provider identifier (NPI) and the TIN of the provider’s group, as well as the claim’s allowed amount, CPT code(s) billed, service date, service location, approval status, and whether Medicare was the primary payer. We restricted our analytic sample to claims from 2009 to 2019 for anesthesia (CPT codes 00100-01999) and emergency medicine (CPT codes 99281-99285, 99291-99292) services. We then excluded any claims where Medicare was not the primary payer, denied claims, claims with non-positive allowed amounts, and claims where the service was not rendered in the 50 states or District of Columbia (i.e., US territories).

This process produced the universe of TINs that billed Medicare for anesthesia or emergency medicine services in each year from 2009-2019, their geographic location(s), the TIN’s total Medicare allowed amounts in a given year (as a measure of the medical group’s size), and the NPIs who billed Medicare under a given TIN. We then incorporated the legal operating name of the TIN, or medical group, from CMS’ Medicare Data on Provider Practice and Specialty (MD-PPAS).

Next, we identified the parent company and ownership structure of TINs through a series of steps.

Because the emergency medicine and anesthesiology markets between 2009 and 2019 experienced a significant number of acquisitions by a relatively small number of companies, we first identified the most active companies making acquisitions using data from Pitchbook and Irving Levin Associates.^[[2]](#footnote-3)^ Some of these companies, themselves, underwent ownership changes during our study period; for instance, Envision, one of the largest staffing companies in both anesthesia and emergency medicine, was publicly-traded in 2009, then brought private by Clayton Dubilier & Rice in 2011, went public again in 2013, and then was brought private again in 2018 by private equity firm Kohlberg Kravis Roberts & Co. (KKR). Therefore, for each of the most active acquirers, we built a timeline of the controlling parent and ownership type in each year. Primarily using data from Pitchbook, we then created a list of each company’s mergers and acquisitions, including those before 2009. This list was supplemented by searches of company websites, social media posts, press releases, and news articles to identify the full set of deals undertaken by the company.

Once all acquisitions by the major players were identified, we matched groups within our analytic dataset to their acquirers and controlling parent companies, largely relying on the TIN’s legal name and geographic location. For example, NorthStar Anesthesia was a large, independent company with groups practicing across multiple markets in 2009 at the start of our study period. All NorthStar-affiliated TINs in our analytic dataset (i.e., NorthStar Anesthesia of Michigan, NorthStar Anesthesia of Pennsylvania, etc.) are coded as part of the same independently owned parent company. Then, in 2013, the company was acquired by private equity firm TPG Growth, jumpstarting a period in which other groups were acquired and brought into the NorthStar Anesthesia umbrella of companies. In our dataset, therefore, all pre-existing NorthStar TINs have their parent company switched to TPG Growth and their ownership type shifted to private equity for 2013 and subsequent years. Companies such as AmSol and Anesthesia Staffing Consultants, which were follow-on acquisitions after the private equity takeover of NorthStar, likewise have their parent company and ownership types for all affiliated TINs switched over in the years they were acquired (2014 and 2015, respectively). When, in 2018, the entire NorthStar Anesthesia family of companies was acquired from TPG Growth by The Cranemere Group, the parent company associated with all NorthStar-affiliated TINs, including those from the follow-on acquisitions, is switched over to the new controlling parent in our dataset. The ownership type remains unchanged following this acquisition, however, since The Cranemere Group is also a private equity firm.

While in some cases matching acquisitions to observations in our analytic dataset was trivial, there are instances in which the TIN legal name field in our analytic dataset did not produce a clean match to an acquired group in Pitchbook. In such cases, we used a combination of practice and business address information, practice owner information, and provider information to find a match between the acquired company in Pitchbook and the associated TIN(s) in the Medicare data.

After this process was complete, a significant portion of the analytic dataset remained unmapped to parent companies and ownership types. This reflects both the fact that private equity and publicly-traded parent companies represent only a fraction of the total market (see Exhibit 1) and that some acquisitions are not captured by Pitchbook or our supplemental searches for acquisitions (which is more common for acquisitions before 2009). As such, we then proceed to map remaining TINs in the dataset to their parent companies and ownership type using data from the CMS National Plan and Provider Enumeration System (NPPES) and articles of incorporation documents from state-level Secretary of State websites. For hospital-owned medical groups, we also grouped TINs into health systems based on American Hospital Association (AHA) and Pitchbook data, such that two medical groups owned by different hospitals in the same health system in the same local market would be considered part of the same parent company when calculating local consolidation levels.

Mapping TINs to parent companies is often trivial using a simple combination of the MD-PPAS organization legal name and Pitchbook data. For example, a group named “Eaton Rapids Medical Center” can be easily matched to an observation in Pitchbook by the same name, which identifies the group as a hospital with no exterior parent company or health system affiliation. Many hospitals and independent groups can be mapped to their parent companies and ownership types using this simple matching process.

It is often the case, however, that the organization legal name in MD-PPAS obscures the ultimate ownership structure of the group practice, particularly for groups owned by private equity or publicly-traded parent companies. For example, “Jackson Drive Emergency Physicians LLC” does not have a match in the Pitchbook data, and preliminary Google searches do not yield a company website or other information about the group. In such cases, we first looked at the NPPES data to identify the authorized official and business address for the TIN. According to the NPPES documentation, an authorized official “must be a general partner, chairman of the board, chief financial officer, chief executive officer, direct owner of 5 percent or more of the provider being enumerated, or must hold a position of similar status and authority within the provider organization,” and has “the legal authority to make changes and/or updates to the provider’s status.” Parent companies typically have a handful of corporate officers who serve as the authorized officials for all constituent groups of their organization and use only a few business mailing addresses for their groups, regardless of the actual practice locations. In the example listed above, the authorized official for the group is the Vice President for Provider Enrollment at Envision, and the business address is listed on the Envision website as one of their corporate offices. Where the NPPES data is more ambiguous in identifying ownership, we looked to articles of incorporation documents for additional confirmatory evidence of parent company ownership.

The NPPES data is also useful for identifying independent groups that share common ownership. Some large, independently-owned practices operate across multiple locations and have numerous distinct TINs that are all constituent parts of the larger parent company. We make use of a similar process that matches authorized officials and business addresses across multiple TINs to ensure we capture common ownership of independent groups, which contributes to the accuracy of our consolidation metrics.

Where uncertainty existed regarding the ultimate controlling entity, we defaulted to assuming a practice is independently owned. Similarly, we faced feasibility constraints on mapping the ownership of every physician group providing emergency medicine or anesthesia services in the country over a more than decade-long period. For groups with less than 0.05% of the national market share for the relevant specialty HCPCS codes and which were not captured by our analysis of acquired groups, we likewise assumed independent ownership.^[[3]](#footnote-4)^ As such, our estimates represent a lower bound on both consolidation and private equity and publicly-traded ownership.

However, benchmarking to public financial statements and media reports bolsters confidence in the accuracy of our crosswalk. For example, in a 2016 SEC filing, TeamHealth states that their target emergency medicine market comprises 2,700 of the 5,000 community hospitals nationwide, and that their market share within the target market is 17%.^[[4]](#footnote-5)^ This would imply that TeamHealth controlled on the order of 9.2% of the national emergency medicine market by their own estimates in 2016, measured as the share of emergency departments staffed. Our own estimates suggest TeamHealth controlled 9.7% of the Medicare emergency medicine service market in 2016, measured based on the share of emergency medicine Medicare allowed amounts billed. Similarly, they state that 2,000 of the 5,000 community hospitals fall in their target anesthesia market, and they have a 3% share within that target market. Using the same rough accounting, they projected that they held about 1.2% of the national anesthesia market in 2016. We estimate that they controlled 0.9% of the Medicare anesthesia service market in that year. It is reasonable to expect small differences given the different methods for measuring market share (not all hospitals bill the same amount of Medicare emergency medicine or anesthesia services).

A Forbes article from March 2018 cites an analyst at Jefferies who studies the healthcare services sector.^[[5]](#footnote-6)^ He estimates that, of the two-thirds of emergency rooms outsourcing their services, Envision controlled 6% of the emergency department and hospital-based physician market, and 7% of the anesthesiology market.^[[6]](#footnote-7)^ While our estimates do not encompass all hospital-based specialties, the cited 7% of the outsourced anesthesia market would come to roughly 4.7% of the total anesthesia market. We estimate they controlled 4.4% of the Medicare anesthesia services market in 2017.

While many other companies were either privately held or did not publicly report estimated market shares, we are also able to use reports of the number of employed physicians to track the accuracy of our crosswalk. Mednax, another publicly-traded firm, reported in their 2017 SEC Form 10-K that they employ 1,400 physicians who provide anesthesia care to patients.^[[7]](#footnote-8)^ Our estimates indicate they had 1,340 anesthesiologists performing services to Medicare patients in that year. Similarly, a January 2016 press release from USAP noted that the company employed “nearly 2,000 providers,” including over 230 in the Denver metropolitan area following an acquisition of South Denver Anesthesiologists.^[[8]](#footnote-9)^ We estimate they had about 1,900 anesthesia providers billing Medicare at the end of 2015, and 228 providers in the Denver HRR at the end of 2016 after the announced acquisition was closed.^[[9]](#footnote-10)^

**Appendix B. Ownership Timelines for Large Anesthesia and Emergency Medicine Staffing Companies**

**
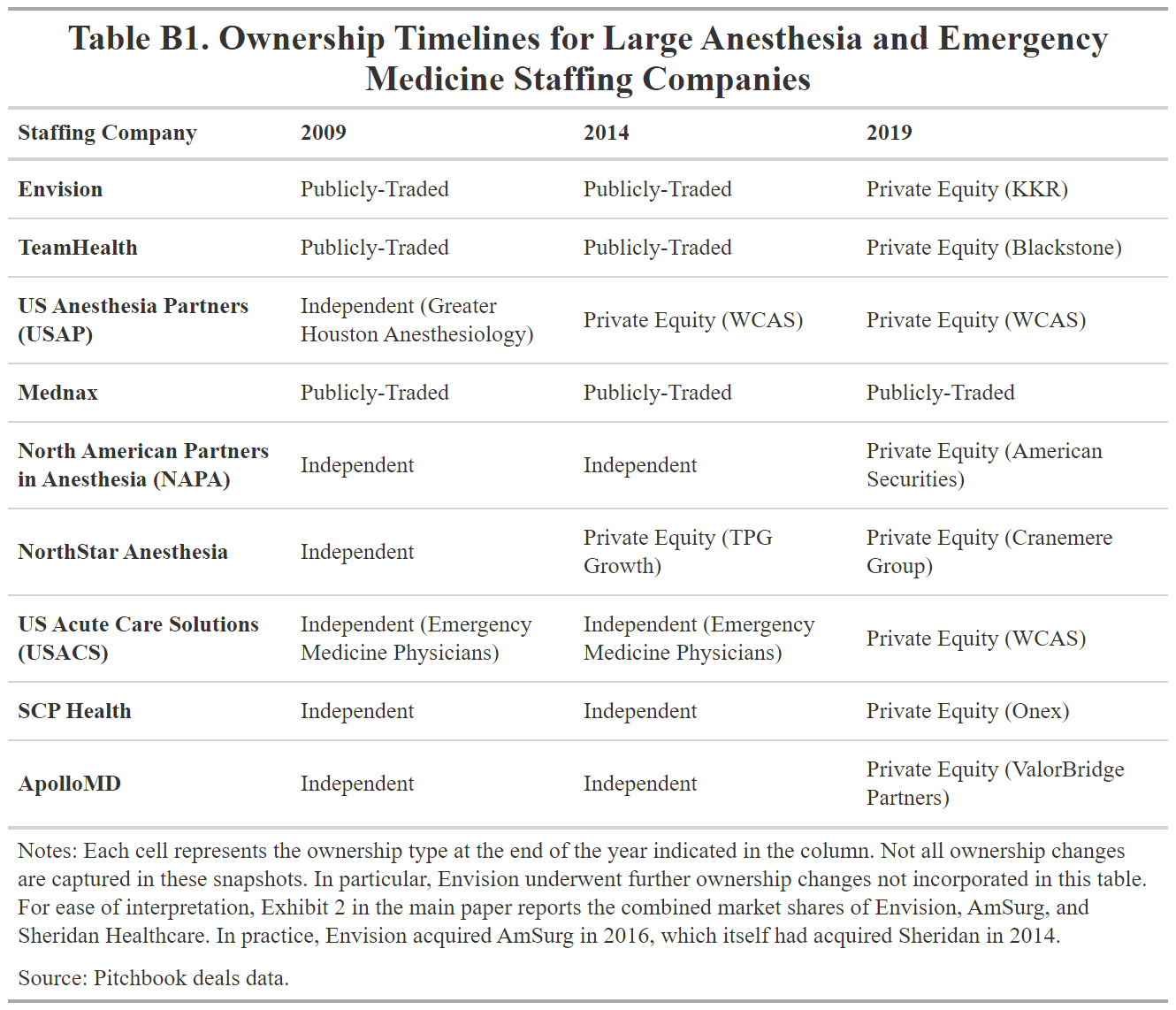
**

**Appendix C. Geographic Variation in Market Concentration Levels, Anesthesiology and Emergency Medicine, 2019**

**
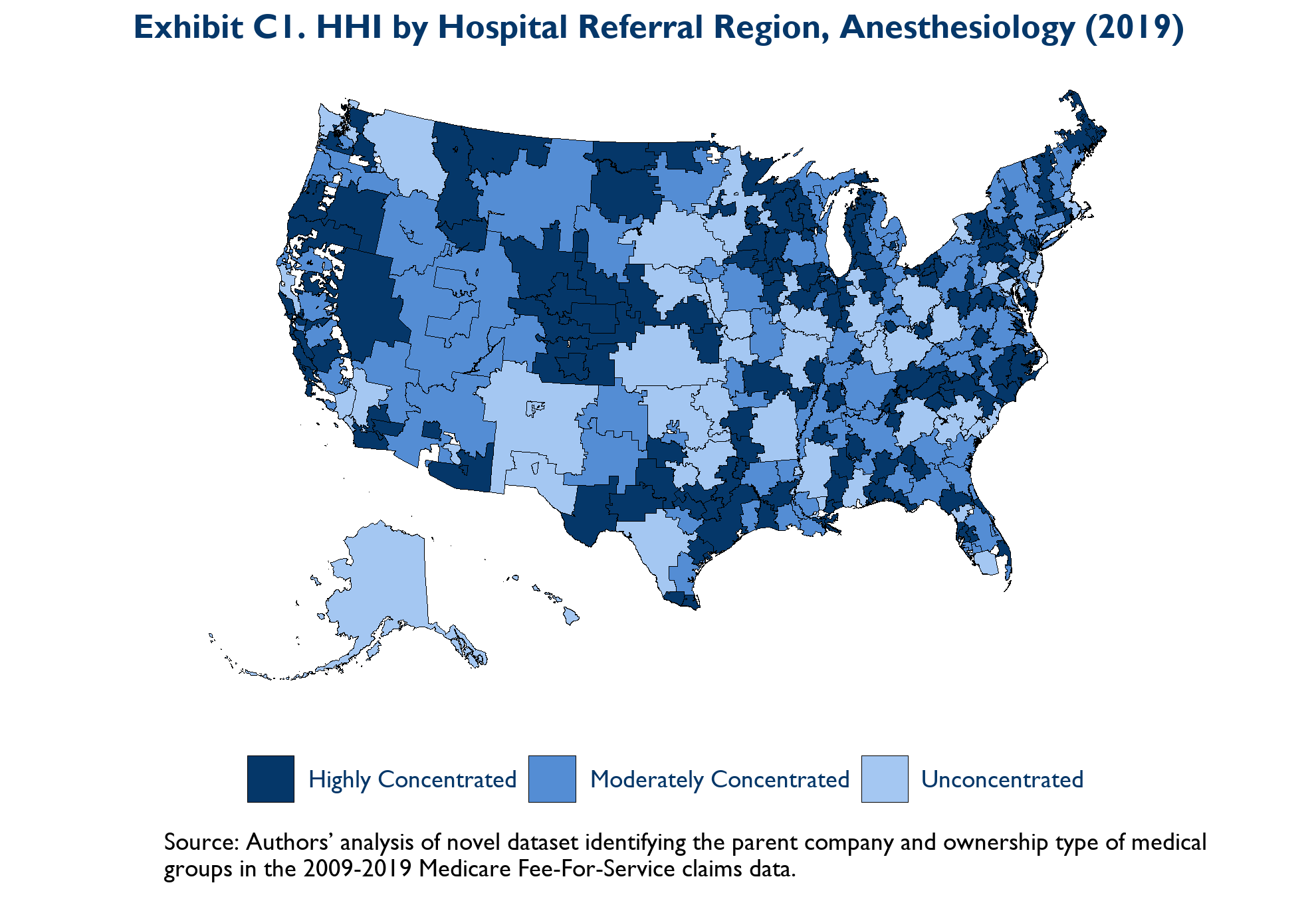
**

**
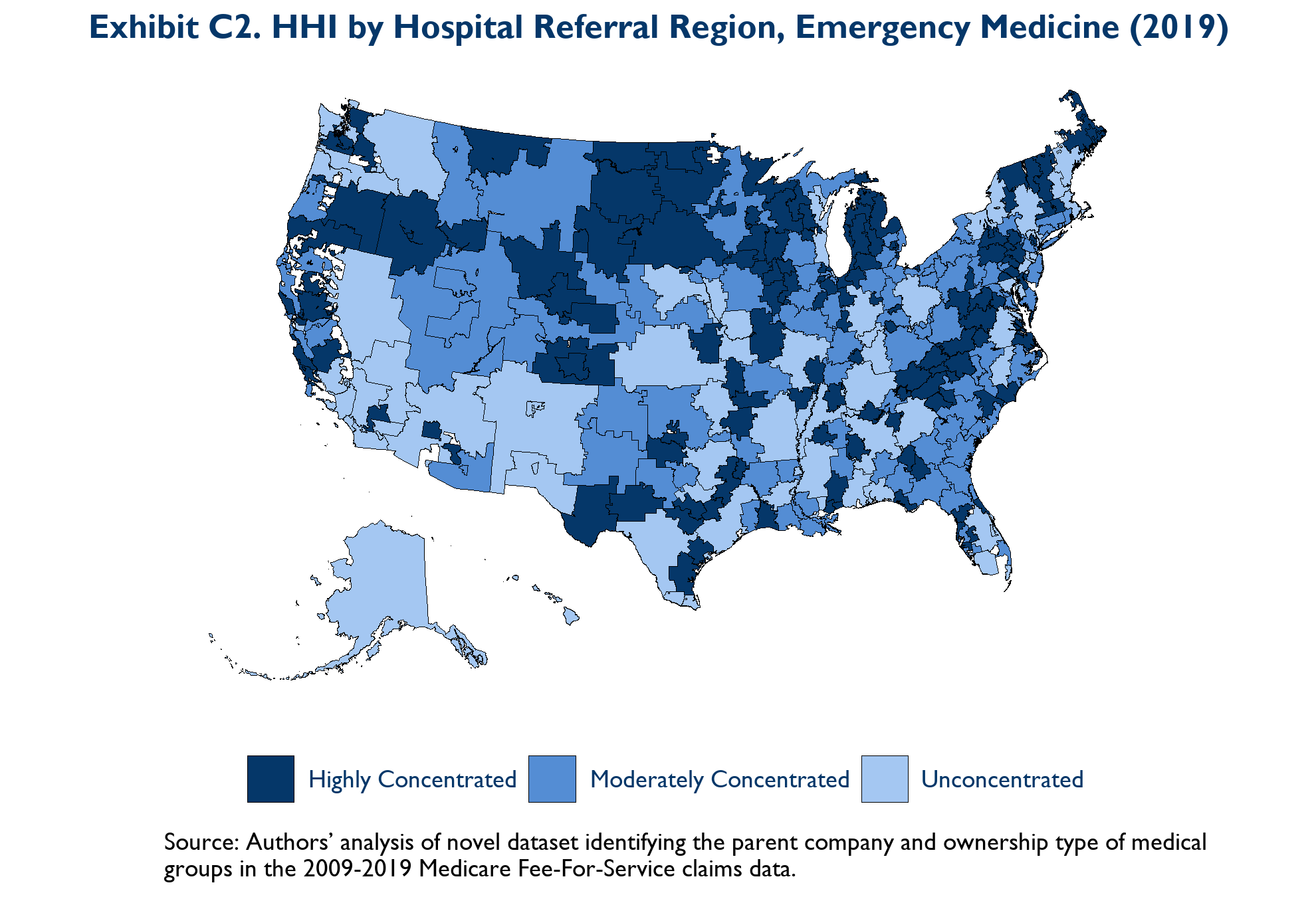
**

**Appendix D. Largest Companies in Highly Consolidated HRRs, 2019
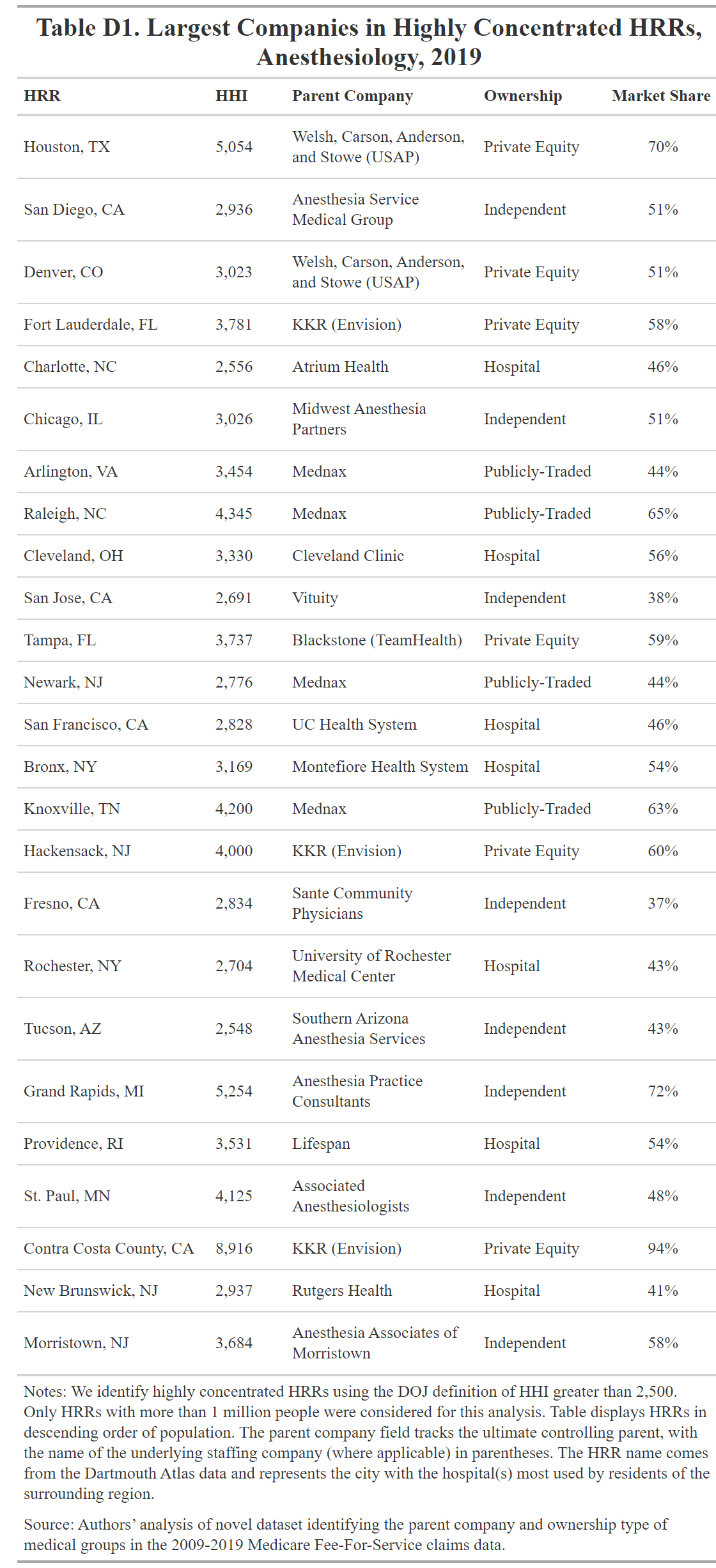
**

**
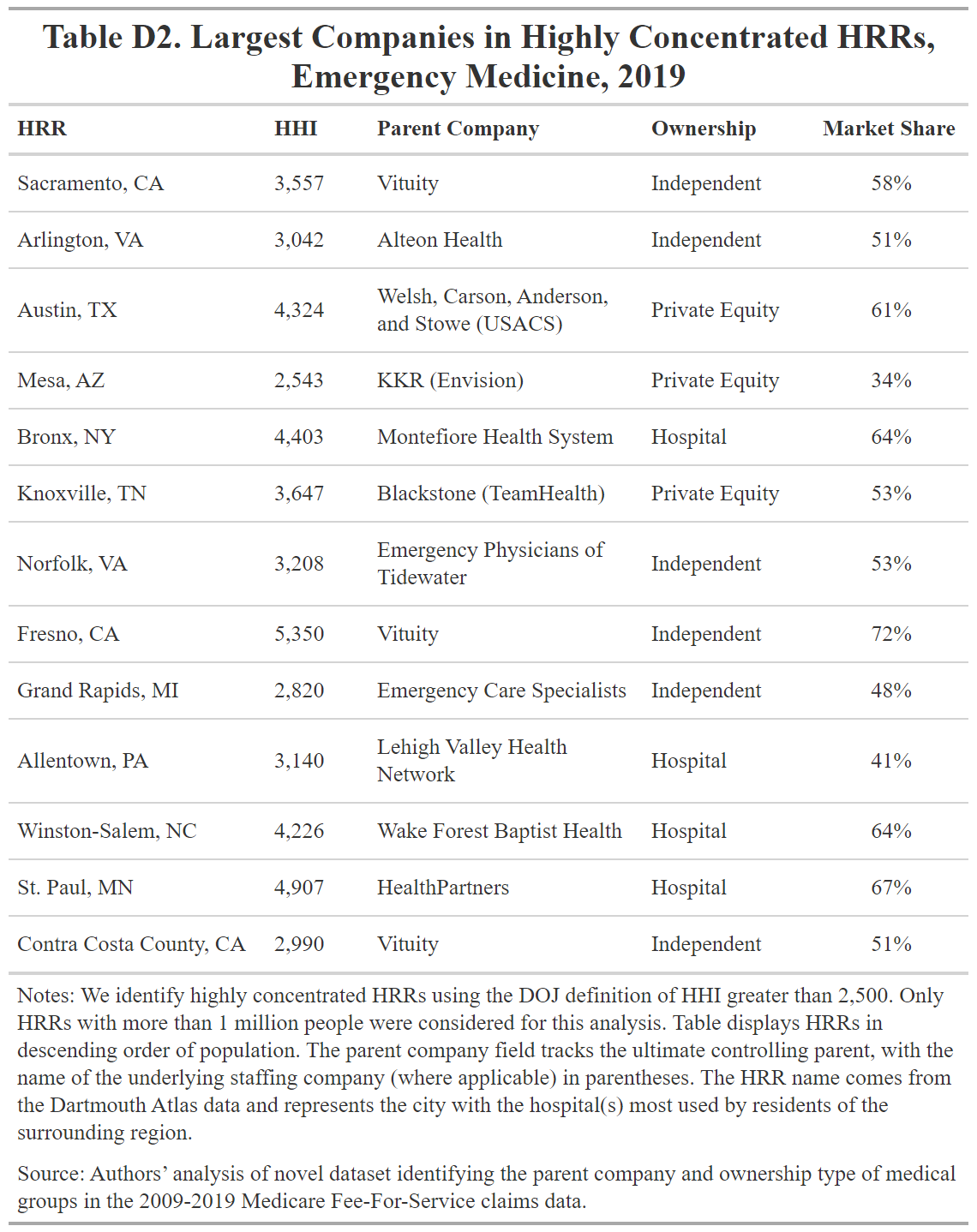
**

1. This is especially true for some of the largest national staffing companies that are owned by private equity and publicly-traded companies. Envision is by far the most active user of this strategy, with more than 200 TINs identified under their ownership as of 2019. TeamHealth and SCP Health likewise employ this strategy, albeit less aggressively, with 83 and 70 TINs mapped to their ownership, respectively. (SCP Health has 70 TINs just for emergency medicine services, as they do not provide anesthesia services. Our estimates for Envision and TeamHealth encompass both specialties.) [↑](#footnote-ref-2)
2. Pitchbook data was accessed via [pitchbook.com](file:///C:\Users\CMilhaupt\AppData\Local\Microsoft\Windows\INetCache\Content.Outlook\A7T0MXZ1\pitchbook.com). Irving Levin data was accessed via [levinassociates.com](https://www.levinassociates.com/). [↑](#footnote-ref-3)
3. It is important to note that using size as a screen leaves our process primarily vulnerable to parent companies who create many small TINs to bill Medicare. As noted above, this is especially true of Envision, TeamHealth, and SCP Health, so our estimates are somewhat more likely to be undercounts for those parent companies. [↑](#footnote-ref-4)
4. The TeamHealth SEC filing was accessed via <https://www.sec.gov/Archives/edgar/data/1082754/000119312517003669/d267388dex991.htm>. [↑](#footnote-ref-5)
5. The estimates from the Forbes article were accessed via <https://www.forbes.com/sites/elliekincaid/2018/05/15/envision-healthcare-infiltrated-americas-ers-now-its-facing-a-backlash/?sh=3f9a9a8d284f>. [↑](#footnote-ref-6)
6. Though the comparison is imperfect, we also estimate hospital ownership of 32% of the Medicare emergency medicine market in 2018, in line with the estimate that roughly two-thirds of emergency rooms outsource their services. [↑](#footnote-ref-7)
7. The Mednax SEC Form 10-K was accessed via <https://mednax.gcs-web.com/static-files/429289a5-ede8-47a9-8304-fd643e16a016>. [↑](#footnote-ref-8)
8. Estimates are drawn from a January 2016 article posted on the USAP website, accessed via <https://www.usap.com/news-and-events/news/us-anesthesia-partners-expands-colorado-through-its-new-partnership-south>. [↑](#footnote-ref-9)
9. These estimates account for all anesthesia service providers, including anesthesiologists, anesthesiology assistants, and certified registered nurse anesthetists. [↑](#footnote-ref-10)
